# Beyond boundaries: fostering women entrepreneurs’ success through culture, family, and entrepreneurship

**DOI:** 10.3389/fsoc.2025.1513345

**Published:** 2025-02-13

**Authors:** Kurniawan, Rival Pahrijal, Agung Maulana, Rakotoarisoa Maminirina Fenitra, Dana Budiman, Supriandi

**Affiliations:** ^1^Department of Management, Universitas Nusa Putra, Sukabumi, Indonesia; ^2^Faculty of Economics and Business, Universitas Indonesia, Depok, Indonesia and ASTA Research Center, Antananarivo, Madagascar; ^3^Department of Management, Telekom University, Bandung, Indonesia

**Keywords:** women entrepreneur, culture, family support, social capital, work-life balance

## Abstract

**Introduction:**

This study investigates how social capital, family support, culture, entrepreneurial qualities, and self-efficacy influence women’s work-life balance and entrepreneurial success in Indonesia.

**Methods:**

This research employs a quantitative methodology, gathering data via a survey with 350 participants.

**Results:**

The investigation findings indicate that culture, family support, and entrepreneurial tendencies significantly influence self-efficacy, social capital, and business success. Additionally, social capital and self-efficacy significantly mediate the association between the independent variables and satisfaction with work-life balance and company performance.

**Discussion:**

The research emphasizes the significance of the interaction between social, cultural, and personal aspects in boosting entrepreneurial success and well-being, and the results offer useful insights to assist the growth of women entrepreneurs in Indonesia.

## 1 Introduction

The number of female entrepreneurs has increased dramatically in recent decades, signaling a significant shift in the direction of gender inclusivity in business ownership and leadership (Wheadon and Duval-Couetil, 2017). This topic is still being discussed in developing nations like Indonesia. Notwithstanding these advancements, systemic impediments and sociocultural limitations still confront women, impeding their ability to pursue entrepreneurial goals (Poulsen et al., 2022). Traditional gender roles, which confine women to the home and limit their potential as corporate leaders, are frequently prescribed by cultural norms and societal expectations (Maxheimer and Nicholls-Nixon, 2022; Sarhan and Ab Aziz, 2023). The persistence of these deeply ingrained beliefs results in unequal access to opportunities, networks, and resources, which impedes women’s advancement as entrepreneurs and slows down economic expansion (Delgado and Murray, 2022). Women’s entrepreneurship faces some obstacles.

Family issues shape women’s goals, motivation, and support systems, which in turn affects their entrepreneurship (McCoy and Winkle-Wagner, 2022). Support from family members can act as a spark, but juggling career and family responsibilities can be difficult (Elotmani and El Boury, 2023). Family support is a cornerstone in the entrepreneurial journey of women, offering a multifaceted and dynamic form of assistance that extends beyond conventional definitions of aid. Emotional encouragement provided by families serves as a critical foundation, instilling confidence and resilience in women entrepreneurs who face the uncertainties and pressures of running a business. Additionally, financial backing from family members often acts as a lifeline, particularly in the early stages of business development when external funding sources may be scarce. Logistical assistance, such as help with managing household responsibilities or providing childcare, further enables women to dedicate more time and energy to their entrepreneurial pursuits. Understanding these dynamics in women’s entrepreneurial journeys is crucial, as demonstrated by the impact of cultural norms and family expectations (Aljarodi et al., 2023; Rametse et al., 2021). Entrepreneurial success depends on both internal and external elements, such as empathy and resilience (Feng et al., 2023; Yu et al., 2024). However, stereotypes and cultural prejudices frequently make it difficult to identify these qualities (Manzoor and Jabeen, 2022; Temitope and Sharma, 2022). To enhance women’s leadership potential, it is critical to have a thorough understanding of how entrepreneurial skills interact with cultural norms (Gerke et al., 2023).

In many cultural contexts, the family’s role transcends passive support to encompass active engagement in business operations, such as offering strategic advice or directly participating in the venture. This symbiotic relationship underscores the interconnectedness between family wellbeing and entrepreneurial success. However, this dynamic is not without challenges. Societal expectations often impose disproportionate caregiving roles on women, creating a delicate balancing act between professional and personal responsibilities. The stress of navigating these dual roles can lead to burnout and hinder entrepreneurial progress, emphasizing the need for systemic solutions and supportive policies.

Furthermore, social capital plays a critical role in the entrepreneurial ecosystem and is particularly important for women entrepreneurs who may encounter additional obstacles when trying to access networks and resources (Sheikh et al., 2021). According to Neumeyer et al. (2019), social capital is the relationships, networks, and connections that support resource mobilization, collaboration, and knowledge sharing within the entrepreneurial community. Several studies further support the idea that social capital is a prerequisite for entrepreneurship. Unfortunately, due to discriminatory behaviors, inadequate participation in business networks, and exclusionary practices, women entrepreneurs frequently experience gaps in the accumulation of social capital (Shankar et al., 2020). This is also the situation for women entrepreneurs in Indonesia. To increase women’s entrepreneurial prospects and promote equitable economic growth, it is imperative to address these disparities and use social capital as a form of empowerment (Mamabolo and Lekoko, 2021; Rana et al., 2022).

Additionally, culture significantly shapes the entrepreneurial ecosystem by influencing societal attitudes, values, and behaviors (Hofstede, 2009). For women entrepreneurs, cultural norms can either serve as facilitators or barriers. In progressive cultures that encourage gender equality, women often have greater opportunities to pursue entrepreneurial ventures. Conversely, in traditional settings, restrictive cultural norms may hinder women’s access to education, resources, and markets. Thus, exploring the intersection of culture and entrepreneurship provides valuable insights into how societal norms can be transformed to support women entrepreneurs. Moreover, self-efficacy, or the belief in one’s ability to achieve specific goals, is a critical psychological factor influencing entrepreneurial behavior (Bandura, 1991). High self-efficacy enables women entrepreneurs to overcome obstacles, make informed decisions, and persist in the face of adversity. It is closely linked to confidence, competence, and a proactive mindset. Furthermore, factors such as mentorship, education, and prior entrepreneurial experience play significant roles in shaping self-efficacy. Understanding how to bolster self-efficacy among women entrepreneurs can lead to more sustainable and impactful ventures.

The ultimate goal of entrepreneurship is often the success and sustainability of the business. Company success is a multidimensional construct encompassing financial performance, market share, customer satisfaction, and social impact. For women entrepreneurs, achieving company success involves navigating a myriad of challenges, from securing funding to competing in male-dominated industries (Reynolds et al., 2024). Additionally, analyzing the factors that contribute to company success provides a roadmap for designing supportive policies and programs tailored to the needs of women entrepreneurs. The intrinsic qualities of entrepreneurs, such as creativity, risk-taking, resilience, and vision, are fundamental to entrepreneurial success. Women entrepreneurs often bring unique perspectives and innovative approaches to business challenges. However, developing and sustaining these entrepreneurial qualities requires a supportive environment that nurtures talent and encourages experimentation. Consequently, identifying the key qualities that distinguish successful women entrepreneurs can help design targeted interventions to foster these traits in aspiring entrepreneurs.

Besides that, balancing entrepreneurial aspirations with personal and family responsibilities is a pervasive challenge for women entrepreneurs. Work-life balance satisfaction is a critical determinant of overall wellbeing and productivity. A harmonious balance enables women to maintain their physical and mental health while achieving their professional goals. However, the lack of institutional support, such as affordable childcare and flexible work arrangements, often exacerbates the work-life balance dilemma. Therefore, investigating the factors that influence work-life balance satisfaction can help identify strategies to enhance the quality of life for women entrepreneurs (O’Hare et al., 2020).

There is still a lack of knowledge about how cultural, familial, and personal factors interact to influence outcomes like business success and work-life balance satisfaction, especially in Indonesia, despite the growing recognition of their significance in shaping women’s entrepreneurial experiences. The majority of studies on women’s self-employment in Indonesia focus on the global context rather than examining the unique dynamics present in Indonesia (Samineni, 2018; Muhaimin et al., 2023). This is concerning because there is an interesting gap where mediating variables, such as self-efficacy and social capital, influence the relationship between independent and dependent variables. Indonesia is distinct in terms of its family values, culture, and socioeconomic issues that impact female entrepreneurs. By investigating the role these elements play in fostering women’s entrepreneurial success in Indonesia, this research aims to close this gap.

The purpose of this study is to elucidate the connections between women’s entrepreneurial success and cultural elements, familial support, entrepreneurial qualities, self-efficacy, and social capital. This study specifically examines three aspects of women’s entrepreneurial success in Indonesia: (Wheadon and Duval-Couetil, 2017) the impact of cultural variables, family support, and entrepreneurial qualities; (Poulsen et al., 2022) the mediating function of self-efficacy and social capital; and (Maxheimer and Nicholls-Nixon, 2022) the relationship between these factors and satisfaction with work-life balance. Comprehending these characteristics is crucial in devising policies and initiatives that foster equitable economic growth, diminish obstacles, and enable female entrepreneurs.

## 2 Literature review and hypothesis development

### 2.1 Theoretical foundation

Entrepreneurship research is deeply rooted in theoretical frameworks that help explain the motivations, behaviors, and outcomes of entrepreneurial activities. Grounding this study in a relevant theoretical foundation enhances its rigor and provides a structured lens through which to explore the dynamics of women entrepreneurs. This study primarily draws upon Social Capital Theory, Resource-Based View (RBV), and Bandura’s Self-Efficacy Theory, while also integrating perspectives from Work-Life Balance Theory and Cultural Theory to provide a comprehensive understanding of the factors influencing women entrepreneurs’ success.

Social Capital Theory (Coleman, 1988; Putnam, 2000) underscores the importance of social networks, relationships, and shared norms in facilitating cooperative action and resource access. For women entrepreneurs, social capital manifests as access to mentorship, funding opportunities, market connections, and collaborative networks. This theory is particularly relevant in exploring how social capital serves as a critical enabler of entrepreneurial success. However, gendered societal norms often present barriers for women in building and leveraging robust social networks, necessitating targeted strategies to enhance social capital for women entrepreneurs.

The Resource-Based View (Barney, 1991) provides a strategic lens for understanding how unique resources and capabilities contribute to sustained competitive advantage. In the context of women entrepreneurship, this framework highlights the significance of tangible resources such as financial capital and technology, as well as intangible assets like entrepreneurial skills, family support, and cultural alignment. By leveraging these resources, women entrepreneurs can achieve superior business performance and long-term sustainability.

Self-efficacy, as proposed by Bandura (1977), is the belief in one’s ability to achieve specific goals and perform tasks effectively. This theory is central to understanding the psychological drivers of entrepreneurial behavior, particularly among women. High self-efficacy enables women entrepreneurs to navigate challenges, take calculated risks, and persist in the face of adversity. Factors such as prior entrepreneurial experience, education, and mentorship are critical in shaping self-efficacy, which in turn influences entrepreneurial outcomes.

Work-Life Balance Theory (Greenhaus and Beutell, 1985) explores the interplay between professional and personal responsibilities. For women entrepreneurs, achieving work-life balance is often a significant challenge, influenced by societal expectations and caregiving roles. This theory helps elucidate how the satisfaction derived from balancing entrepreneurial pursuits with personal wellbeing impacts overall success and quality of life. A better understanding of work-life balance dynamics can inform policies and practices that support women entrepreneurs in managing these dual responsibilities.

Hofstede’s Cultural Dimensions Theory (Hofstede, 2001) provides a framework for analyzing how cultural norms and values shape entrepreneurial behavior. Women entrepreneurs operate within diverse cultural contexts that can either facilitate or hinder their ventures. For instance, progressive cultures that emphasize gender equality tend to provide more opportunities for women entrepreneurs, whereas traditional cultures may impose restrictive norms. This theory allows for a nuanced exploration of how cultural factors intersect with entrepreneurship and influence success.

The integration of these theoretical perspectives provides a robust foundation for examining the multifaceted nature of women entrepreneurship. Social Capital Theory and RBV emphasize the importance of networks and resources, while Self-Efficacy Theory addresses the psychological dimensions of entrepreneurial behavior. Work-Life Balance Theory and Cultural Theory add layers of complexity by highlighting the socio-cultural and personal challenges faced by women entrepreneurs. These theories create a comprehensive framework for understanding the variables influencing women entrepreneurs’ success and identifying actionable strategies to support them.

### 2.2 Culture

Through socialization, beliefs, values, rituals, behaviors, and artifacts are passed down from generation to generation, forming culture and influencing how people perceive and engage with their surroundings. As new concepts arise and traditional customs alter, culture keeps changing (Nadkarni and Prügl, 2021). According to Hofstede’s cultural dimensions, cultural traits like indulgence against restraint, power distance, individuality versus collectivism, masculinity versus femininity, long-term versus short-term orientation, and individualism versus collectivism all have a significant impact on how organizations run. In addition to posing possibilities and problems in relationships with clients, staff, and rival businesses, these aspects also shape organizational values, attitudes, and practices (Hofstede, 2009; Migon Favaretto et al., 2019).

Culture affects corporate strategy and cross-cultural communication in different nations, and it has a major impact on several areas of personal and professional life, including work-life balance, social capital, self-efficacy, and company performance (Bullough et al., 2022). There are now clear distinctions between national and international entrepreneurial cultures as a result of the shift from a Soviet-style economy to a market-based economy, especially in Russia (Kuznetsov and Kuznetsova, 2005). Cultural aspects impact company strategies and marketing management in South Asia. These elements include the significance of adapting to local cultural nuances and maintaining a market orientation (Kotler and Keller, 2009). The complexity of cross-cultural business communication is also rising as a result of regional variations in norms, expectations, and language usage. To thrive internationally, organizations must forge strong ethical and cultural identities (Beckers and Bsat, 2014). National cultures have an impact on self-efficacy, risk-taking, social capital, and organizational and individual adaptability—all of which are critical for business success in an international setting. The body of current literature offers proof and generates hypotheses:

*H1*: There is a significant positive influence of culture on self-efficacy in Indonesian women entrepreneurs.

*H2*: There is a positive and significant effect of culture on social capital in Indonesian women entrepreneurs.

*H3*: There is a positive and significant influence of culture on business success in female entrepreneurs in Indonesia.

*H4*: There is a positive and significant influence of culture on work-life balance satisfaction in women entrepreneurs in Indonesia.

### 2.3 Family support

Family support, which is crucial to the wellbeing of the person and the family, consists of practical, financial, and emotional support (Marier, 2021; Blass and Shelah, 1989). This assistance—which could take the form of listening, giving money, or helping with everyday duties—contributes to the stability of the family (Scheff, 2014). According to Mayes et al. (2022), social capital, which consists of networks and norms, is just as valuable as family support (Falk and Harrison, 1998; Schröder et al., 2020) when it comes to coping with socioeconomic change. Although obstacles like miscommunication and expectation gaps might occur, this support helps entrepreneurs succeed in many ways (Arif and Hamid, 2023). Family support for entrepreneurs has been linked to increased motivation, success, and decreased stress levels (Hasanah et al., 2022).

Family support contributes to security, development, and resilience, which are critical components of company success, particularly for family-owned enterprises. Family businesses are more likely to be stable, maintain business continuity, and assist in overcoming obstacles at different stages of development, according to research (Memili et al., 2023). Additionally, this support builds social capital, which aids in knowledge sharing between families and communities and supports small enterprises in overcoming adversity. Furthermore, family support helps people overcome obstacles and provide resources for success, which has an impact on people’s careers, particularly in demanding fields like professional kitchens (Spieß et al., 2022) and minority-owned enterprises like refugee-owned businesses (Holland and Oliver, 1992; Torres and Marshall, 2015; Joseph, 2022).

Work-life balance satisfaction is significantly influenced by family support (Khalid et al., 2023). WLB is highly influenced by supervisor support and a flexible work environment (Rahmansyah et al., 2023; Yus et al., 1974; Banik et al., 2021). Furthermore, extended family serves as a vital source of social support, particularly for moms who work (Uddin et al., 2022). Work-family balance decisions are also influenced by cultural and policy considerations (Banik et al., 2021; Regina et al., 2021). Role overload can be lessened and WLB’s efficacy can be increased with the assistance of colleagues and the organization (Ninaus et al., 2021; Boakye et al., 2021). To increase employee wellbeing and productivity, organizations should adopt family-friendly policies (Akobo and Stewart, 2020; Giao et al., 2020; Chatra and Fahmy, 2018). The body of current literature offers proof and suggests theories.

*H5*: There is a positive and significant effect of family support on self-efficacy.

*H6*: There is a positive and significant effect of family support on social capital.

*H7*: There is a positive and significant effect of family support on business success.

*H8*: There is a positive and significant effect of family support on work-life balance satisfaction.

### 2.4 Entrepreneurial traits

The success of a firm depends on having entrepreneurial qualities including vision, confidence, risk-taking, inventiveness, and resilience. People who solve problems creatively, take calculated chances, and learn from mistakes are viewed as entrepreneurs. Their ability to innovate allows them to remain competitive and adjust to changes in the market (Nambisan, 2017; Toma et al., 2014). The capacity to bounce back from setbacks and make sound business decisions is influenced by risk-taking and resilience (Dees, 1998; Zahra and Wright, 2016). Successful entrepreneurship is also favored by effective leadership and financial and personal motivation (Shukla, 2021; Iskandar et al., 2021). Extant literature supports the following hypothesis and offers evidence for it:

*H9*: There is a positive and significant effect of entrepreneurial traits on self-efficacy.

*H10*: There is a positive and significant effect of entrepreneurial traits on social capital.

*H11*: There is a positive and significant effect of entrepreneurial traits on business success.

*H12*: There is a positive and significant influence of entrepreneurial traits on work-life balance. Satisfaction.

### 2.5 Mediators of motivation, personal characteristics, and new venture performance

The interplay of various cognitive, behavioral, and psychological traits shapes entrepreneurial success. The relevance of heuristics and biases in decision-making is highlighted by cognitive techniques, particularly when faced with uncertainty and novelty. The Five Factor Model’s description of personality traits has been connected to entrepreneurial success; digital entrepreneurs, for instance, tend to employ neuroticism to their advantage (Alomani et al., 2022). According to Bandera and Passerini (2018), the interplay among human, social, and cognitive resources is essential for the success of budding entrepreneurs. Numerous studies demonstrate that a variety of entrepreneurial traits, such as demographic variables, can account for a significant portion of the expansion of small and medium-sized businesses (SMEs) (Thinji and Gichira, 2017). Researchers and entrepreneurs alike can benefit from a greater understanding of these variables as they pertain to the dynamics of business success and entrepreneurship.

According to Albert Bandura, self-efficacy—a person’s confidence in their capacity to complete a job or reach a goal—is a crucial component of motivation and behavior modification. In domains like entrepreneurship, education, and parenthood, self-efficacy affects emotions, problem-solving, and overall wellbeing (Hvalič-Touzery et al., 2022; Karvonen et al., 2023; Maghfiroh et al., 2023; Poluektova et al., 2023; Sehlström et al., 2023). Studies reveal that self-efficacy promotes optimism and learning engagement (Optimism, 2017) and connects favorably with competence and negatively with reading anxiety (Trisnayanti et al., 2020). According to Hodges et al. (2014), teacher self-efficacy promotes the effectiveness of new curricula in the classroom and influences health-related behaviors in the domain of physical fitness.

Studies reveal that self-efficacy influences employee entrepreneurial behavior (Kim and Beehr, 2023), mediates the relationship between social capital and entrepreneurial intention (Mahfud et al., 2020), and moderates the relationship between financial literacy and SME sustainability (Julito et al., 2021). Gender differences also exist in entrepreneurial self-efficacy, with males reporting higher levels than women (Nanjala, 2012). According to a study on coach and parent support in Malaysia, self-efficacy development is favorably correlated with work-life balance satisfaction (Ketelle, 2005; Retnam et al., 2018). Emotional intelligence and job stress have an impact on self-efficacy as well (Wilson, 2016; Soloman, 2014; Olokoyo et al., 2009). The body of current literature supports the following hypothesis and offers evidence:

*H13*: There is a positive and significant influence of self-efficacy on business success.

*H14*: There is a positive and significant effect of self-efficacy on work-life balance satisfaction.

The term “social capital” describes the value that a community places on shared norms, social ties, and trust—all of which have the potential to enhance both individual and societal wellbeing. Social capital affects a range of social phenomena, including quality of life, sustainable agriculture, and catastrophe mitigation. It also encompasses the advantages of relationships, such as access to resources, information, and support (Purba et al., 2022; Nugraha et al., 2020). But social capital can also have a dark side that could be detrimental to society; this is known as “dark social capital.” Relationships between people, groups, and institutions are impacted by this idea, as seen in the producer-supplier relationship (Nugraha et al., 2020; Mungra and Yadav, 2023). Indicators including social awareness, participation, trust, and reciprocity are used to quantify social capital. Research conducted in China revealed a connection between social capital and religious inclinations, whereas studies conducted in Egypt employed 12 social capital variables for rural populations (Newaser and Basha, 2023). In addition, social capital affects adult population health in Norway (Gele and Harsløf, 2010) and the ability of small enterprises to bounce back from natural disasters (Norris, 2007).

Studies indicate that work-life balance satisfaction is positively impacted by social capital, as people can manage their personal and professional obligations with the assistance of coworkers, managers, and flexible scheduling (Ghodsee and Orenstein, 2021; Duffy and Dik, 2009; Wilkinson, 2014). In addition to lowering stress and enhancing wellbeing, social capital also gives people access to information and emotional support; nevertheless, the impacts of social capital might vary depending on personal traits and socioeconomic circumstances (Bartolini and Sarracino, 2015). Furthermore, trust, resource accessibility, and small business resilience following disasters are all influenced by social capital, which is crucial for business success (Torres and Marshall, 2015; Meiryani et al., 2023). Social capital is a significant component of business and entrepreneurship (Colfax et al., 2010). It also promotes entrepreneurial ambitions through self-efficacy (Mahfud et al., 2020) and entrepreneur performance (Pullich, 2012). The body of current literature supports the following hypothesis and offers evidence:

*H15*: There is a positive and significant effect of social capital on business success.

*H16*: There is a positive and significant influence of social capital on work-life balance satisfaction.

### 2.6 Key factors business success and work life balance satisfaction

A broad spectrum of operational, financial, and strategic accomplishments are components of business success (Tiwari and Suresha, 2021). It is determined by how well the business meets or surpasses its objectives, pleases clients, and experiences sustainable growth (Alabdullah, 2023). Quantitative measurements, including financial performance that includes revenue growth, profitability, return on investment (ROI), and cash flow, can be used to evaluate success indicators (Dimitropoulos and Scafarto, 2021). Strong financial performance is typically seen as a successful business (Jylhä et al., 2020), as demonstrated by the consistent growth in revenue and profitability. However financial performance on its own is insufficient (Prentice, 2022). A company’s performance can also be determined by factors such as employee engagement, customer happiness, and brand reputation (Sánchez-Iglesias et al., 2024). Employee involvement boosts productivity and creativity, while customer happiness leads to referrals and repeat business (Capelle, 2013). Customers, employees, and investors are drawn to brands with a good reputation (Boikanyo and Naidoo, 2023). According to Vibhakar et al. (2023), business success is a multifaceted notion that encompasses both financial and operational metrics. The company’s total success is determined by the performance of both of these areas.

**Figure 1:**
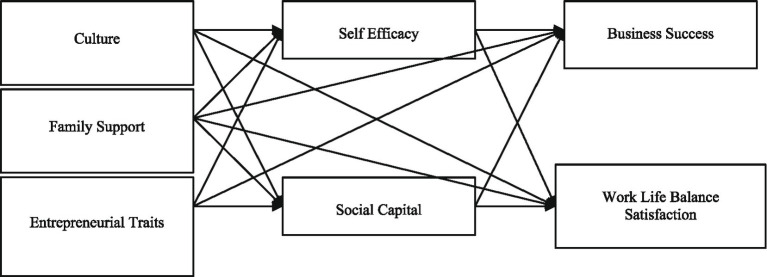
Research framework.

A person’s attempts to strike a balance between their personal and professional obligations are referred to as work-life balance (Shanafelt et al., 2012). It entails time and energy management to ensure that relationships, leisure activities, and personal wellbeing are not sacrificed to fulfill job obligations (Shirmohammadi et al., 2022). This balance, which enables one to pursue a job while still having time for family, hobbies, and self-care, is crucial for happiness, productivity, and mental health (Vyas, 2022; Susanto et al., 2022). Setting limits, prioritizing work, and scheduling downtime are necessary to achieve it (Azapagic and Perdan, 2000). Time spent on work vs. leisure activities, stress levels, health, and life satisfaction are all indicators of this balance (Budhiraja et al., 2022). Better balance is also facilitated by flexible work schedules and the freedom to put work on hold after hours (Jaharuddin and Zainol, 2019; Opatrná and Prochazka, 2023). A research model framework is developed based on the accumulated literature, as illustrated in Figure 1.

## 3 Methods

### 3.1 Participants

This study focused exclusively on women entrepreneurs, with the sample selected using a combination of simple random sampling, non-probability sampling, and purposive sampling techniques. The rationale for employing multiple sampling methods lies in the study’s objectives and the practical constraints faced during data collection.

Simple random sampling was used in the initial phase of the study to ensure that every individual in the defined population of women entrepreneurs had an equal probability of being selected. This approach was particularly useful in maintaining the objectivity and representativeness of the sample. For instance, when reaching out to registered women entrepreneurs through formal business networks or government listings, a random selection process was conducted to prevent selection bias and ensure diversity in the sample.

Non-probability sampling was then integrated to address logistical constraints and practical challenges, such as accessing informal or less-visible women entrepreneurs who might not be part of formal networks. This method proved effective for recruiting participants in online surveys, where respondents were reached through social media platforms, online forums, and community groups. Non-probability sampling allowed the study to engage with women entrepreneurs who may not have been captured through traditional random sampling, broadening the scope of the research.

Purposive sampling played a critical role in ensuring that the sample included participants who met specific criteria aligned with the study’s objectives. The purposive criteria included women entrepreneurs who were actively managing their businesses, had a minimum of 2 years of entrepreneurial experience, and operated within various sectors such as retail, services, and manufacturing. This targeted approach ensured that the sample reflected the nuanced perspectives of experienced women entrepreneurs, enabling a deeper exploration of the research variables.

To operationalize these methods, the study distributed 350 questionnaires over a three-month period using both offline and online channels. Offline distribution involved face-to-face engagement during networking events, entrepreneurial workshops, and visits to business locations. Enumerators approached participants identified through random and purposive selection processes, ensuring that responses represented a mix of urban and rural entrepreneurs. Meanwhile, online distribution leveraged platforms like LinkedIn, Facebook, and WhatsApp groups to circulate the survey, particularly among informal or remote entrepreneurs who were otherwise difficult to reach.

Thanks to the diligent efforts of the author and enumerators, all 350 questionnaires were successfully collected. While this high response rate is commendable, it is essential to acknowledge that combining multiple sampling methods may introduce complexity and potential inconsistencies if not systematically justified. In this study, the chosen techniques were employed to strike a balance between representativeness, inclusivity, and practical feasibility.

### 3.2 Research design

To produce numerical data and examine the link between the variables, this study adopted a quantitative technique. A structured survey that was disseminated electronically via email, Facebook, LinkedIn, Instagram, and other social media platforms was used to gather data.

### 3.3 Measurement

Table 1 shows the measurement used in this model, all the items were adopted from previous studies and modify to adjust within the present context.

**Table 1:** Measurement.

Variable	Items	Code	Source
Culture	In my culture, it is important to respect authority figures such as parents, teachers, and bosses.	CLT.1	Hofstede (2009) and Migon Favaretto et al. (2019)
	In my culture, there is a strong emphasis on planning and preparation to minimize uncertainty.	CLT.2	
	In my culture, people are expected to prioritize the needs of the group over their individual desires.	CLT.3	
	In my culture, assertiveness and competitiveness are traits that are highly valued in men.	CLT.4	
	In my culture, nurturing and caring behaviors are equally valued in both men and women.	CLT.5	
	Immediate gratification is prioritized over long-term benefits in my culture.	CLT.6	
	People in my culture believe in controlling their desires and impulses for the greater good	CLT.7	
Family support	I feel comfortable discussing my feelings and emotions with my family members.	FMS.1	Holland and Oliver (1992), Torres and Marshall (2015), and Joseph (2022)
	My family is willing to provide financial assistance to me in times of need.	FMS.2	
	My family is proactive in offering practical assistance whenever necessary	FMS.3	
	I can leverage my family’s social connections to access opportunities or resources.	FMS.4	
	My family members are skilled at resolving conflicts in a constructive and respectful manner.	FMS.5	
Entrepreneurial trait	I am always eager to try new things and explore innovative ideas.	ETT.1	Toma et al. (2014) and Nambisan (2017)
	I am diligent and detail-oriented when it comes to completing tasks and achieving goals.	ETT.2	
	I feel energized and motivated when interacting with others, and I actively seek out networking opportunities.	ETT.3	
	I value collaboration and teamwork, and I strive to maintain positive relationships with others in my professional endeavors.	ETT.4	
	I tend to stay calm and composed even in stressful situations, allowing me to make rational decisions.	ETT.5	
Self-efficacy	I believe that I am capable of achieving high grades in my academic pursuits.	SEF.1	Hvalič-Touzery et al. (2022), Karvonen et al. (2023), Maghfiroh et al. (2023), Poluektova et al. (2023), and Sehlström et al. (2023)
	I am confident that I can effectively manage my study time and resources to achieve academic success.	SEF.2	
	I am confident in my ability to maintain a regular exercise routine and improve my physical health.	SEF.3	
Social capital	I am attentive to social dynamics and cues in various social situations, which helps me navigate relationships effectively.	SCP.1	Purba et al. (2022) and Nugraha et al. (2020)
	I actively engage in community events, gatherings, and activities to connect with others and contribute to the community.	SCP.2	
	I trust the people in my social network to provide support, guidance, and assistance when needed.	SCP.3	
	I am willing to offer help and support to others in my social network, expecting that they will reciprocate when I need assistance.	SCP.4	
Business success	I measure business success by the consistent growth in revenue over time.	BSC.1	Dimitropoulos and Scafarto (2021), Jylhä et al. (2020), and Prentice (2022)
	Maintaining a healthy profit margin is crucial for assessing the success of my business.	BSC.2	
	I consider the return on investment (ROI) to be a critical metric for evaluating the success of business initiatives.	BSC.3	
	A steady and positive cash flow is indicative of a successful and sustainable business.	BSC.4	
	Building long-term relationships with satisfied customers is crucial for the sustained success of my business.	BSC.5	
	I prioritize creating a positive work environment that fosters employee satisfaction and engagement.	BSC.6	
	Maintaining a strong and positive brand reputation is essential for the success and longevity of my business.	BSC.7	
Work life balance	I feel that I have a good balance between the time I dedicate to work and the time I allocate for personal activities.	WLB.1	Vyas (2022) and Susanto et al. (2022)
	My work-life balance positively impacts my stress levels, allowing me to manage challenges effectively.	WLB.2	
	I prioritize both my physical and mental health, and I believe that maintaining a good work-life balance contributes to my overall wellbeing.	WLB.3	
	I am satisfied with my overall quality of life, which includes factors such as work, relationships, and personal pursuits.	WLB.4	

Source: literature review.

### 3.4 Data analysis

The gathered data examined using SEM-PLS path analysis with the aid of SmartPLS, enabling the simultaneous estimate of measurement and structural models (Vibhakar et al., 2023). Confirmatory factor analysis (CFA) was used to assess the variance inflation factors (VIF), reliability, discriminant validity, and convergent validity (Shanafelt et al., 2012). The convergent validity assessed using factor loadings, composite reliability (CR), and average variance extracted (AVE); the discriminant validity assessed using the HTMT ratio and the Fornell-Larcker criterion (Shirmohammadi et al., 2022). Reliability assessed using Cronbach’s alpha and composite reliability, with data reliability being ensured via VIF (Vibhakar et al., 2023). We’ll utilize bootstrapping with 5,000 resamplings to generate confidence intervals and standard errors. Indexes like goodness-of-fit (GoF) and normed fit index (NFI) are used to evaluate the fit of a model (Shanafelt et al., 2012). The results of the link between latent constructs and observable variables shows in a path diagram, along with path coefficients, *t*-values, *p*-values, and R-squared values to show how significant and strong the association is (Vibhakar et al., 2023) (Table 1).

## 4 Results and discussion

### 4.1 Respondent demographics

According to Hair et al. (2017), samples in SEM-PLS should be aware of missing data outliers; however, in this study, all questions were answered by 100% of respondents, and there were no missing data outliers. In addition, it is recommended that the research indicators be multiplied by five or 10. In this study, there are 35 indicators multiplied by 10, which means that 350 samples are considered relevant to the recommendation (Hair et al., 2017). The age represented the following: 25% of the responding population came from Java, 30% from Sumatra, 18% from Kalimantan, and 27% were from Sulawesi. About 21% of the responding population was born Javanese, 31% were Sundanese, 19% were Balinese, and the remaining 29% had other roots. For education, 22% of respondents completed junior high school, 20% completed senior high school, 32% completed a bachelor’s degree, and 26% obtained a master’s or doctoral degree. Business experience is also diverse among the respondents: <10 years, 29%; between 10 and 15 years, 24%; between 15 and 20 years, 28%; and above 20 years, 19%. The technology sector represents 4%, manufacturing 25%, retail 23%, agriculture 18%, and the other industry sectors are 30% represented. By ownership structure, 23% of company ownership is owned by an individual or a family, 27% are CVs, 20% are limited liability companies, while the remaining 30% falls under other ownership structure categories. Of these, in terms of workers, 25% have less than five people, 29% between five and 20 people, 20% between 20 and 100 people, while 26% have more than 100 people.

### 4.2 PLS SEM requirements

This research employs confirmatory factor analysis (CFA), which is based on sound theory, to ensure that the model is robust and that the latent variable indicators are adequate. In the PLS-SEM technique, outer model analysis is used to determine the construct validity and reliability. The feedback forms were used to check the validity and reliability of data, and specialists were used to validate the data. In this study, the VIF, CR, CA, HTMT ratio, and AVE were assessed. All the values were >0.70, thus showing that the measuring scale was sufficiently reliable (Hair et al., 2017). From the results, there are no multicollinearity issues since the AVE is >0.50 and the outer VIF is less than the threshold of 3 (Hair et al., 2017). All the results for each item were satisfactory (Table 2).

**Table 2:** PLS requirements test.

Variable	Item	OL	CR	CA	AVE	VIF
Culture	CLT.1 CLT.2 CLT.3 CLT.4 CLT.5 CLT.6 CLT.7	0.801 0.792 0.851 0.811 0.901 0.812 0.785	0.899	0.788	0.765	2.450 2.854 2.678 3.876 2.989 2.001 2.984
Family support	FMS.1 FMS.2 FMS.3 FMS.4 FMS.5	0.872 0.914 0.845 0.822 0.915	0.922	0.893	0.789	2.006 2.854 2.650 2.448 2.206
Entrepreneurial traits	ETT.1 ETT.2 ETT.3 ETT.4 ETT.5	0.827 0.900 0.821 0.797 0.806	0.904	0.875	0.756	2.201 2.905 2.476 2.998 2.761
Self-efficacy	SEF.1 SEF.2 SEF.3	0.901 0.896 0.791	0.885	0.894	0.876	2.000 2.903 2.875
Social capital	SCP.1 SCP.2 SCP.3 SCP.4	0.899 0.806 0.864 0.871	0.900	0.900	0.865	2.523 2.980 2.880 1.069
Business success	BSC.1 BSC.2 BSC.3 BSC.4 BSC.5 BSC.6 BSC.7	0.922 0.874 0.844 0.798 0.895 0.867 0.920	0.899	0.874	0.843	2.447 1.098 2.146 2.144 2.067 2.986 2.516
Work life balance	WLB.1 WLB.2 WLB.3 WLB.4	0.899 0.925 0.876 0.988	0.871	0.883	0.795	2.045 2.051 2.751 1.004

Source: data analysis result, 2024.

Validity of the model was used to measure factors such as culture, work-life balance, entrepreneurial traits, self-efficacy, family support, and social capital in regard to entrepreneurship and business success. Evaluation of each construct was measured by AVE, VIF, Cronbach’s Alpha, composite reliability, and outer loadings (Henseler et al., 2015). While internal consistency with values above 0.70 is demonstrated by composite reliability and Cronbach’s Alpha, outside loadings indicate the intensity of the association between items and constructs (Shirmohammadi et al., 2022). Convergent validity is assessed through AVE while multicollinearity is assessed through VIF (see below 3) (Hair et al., 2019).

Table 3 multicollinearity and discriminant validity between variables. The square root of AVE on the diagonal is higher than the correlation coefficient off the diagonal, showing strong discriminant validity (Henseler et al., 2015). The VIF score shows low multicollinearity among predictor variables, hence making the study results more valid and reliable for analysis and interpretation (Hair et al., 2019).

**Table 3:** Discriminant validity and inner VIF values.

Variable	CLT	FMS	BSC	SEF	ETT	SCP	WLB
Discriminant validity							
Culture							
Family support	0.562						
Entrepreneurial traits	0.341	0.312					
Self-efficacy	0.461	0.500	0.368				
Social capital	0.718	0.272	0.383	0.165			
Business success	0.626	0. 381	0.266	0.570	0.603		
Work life balance	0.656	0.619	0.185	0.283	0.400	0.605	
Inner VIF							
Culture			2.216	2.095		2.004	2.045
Family support			1.528	1.988		1.769	2.007
Entrepreneurial traits			2.433	2.901		2.901	1.984
Self-efficacy			3.012				2.013
Social capital			4.127				2.264

Source: data analysis result, 2024.

SRMR, d_ULS, d_G, chi-square, and NFI are model fit criteria against the saturated model. Generally speaking, the estimated model is closer to the data and thus better. The d_ULS and d_G were greater, while the SRMR was low, 0.035 compared to 0.038 for the saturated model. The chi-square was a bit higher for the estimated model; however, the NFI displayed a higher model fit. These results imply that the model estimated fits with the data better (Hair et al., 2019) (Table 4).

**Table 4:** Model fit criteria.

	Saturated model	Estimated model
SRMR	0.038	0.035
d_ULS	0.462	0.518
d_G	0.394	0.397
Chi-square	2794.183	2852.133
NFI	0.803	0.827

Source: data analysis result, 2024.

### 4.3 Structural model test result

When exogenous variables changes cause an effect on endogenous variables, the magnitude of path coefficient, or standardized beta (*β*), is estimated by the PLS-SEM method. Paths with large values reflect strong influence, while paths with tiny values reflect a weak impact. If the t-statistic is >1.96 at 95% confidence level, the hypothesis is significant (Hair et al., 2019). These results were achieved with SmartPLS bootstrapping. Using a 0.05 *p*-value as the decision reference, the following Table 5 presents the hypothesis analysis with beta value, means, standard deviations, *t*-values, and *p*-values.

**Table 5:** Structural model test result.

Path	Coefficient	*T*-value	*p*-value	Hypothesis checking
Direct effect				
CLT → SEF	0.184	0.778	0.037	Accepted
CLT → SCP	0.208	1.896	0.041	Accepted
CLT → BCS	0.307	3.089	0.002	Accepted
CLT → WLB	0.325	1.340	0.023	Accepted
FMS → SEF	0.452	4.157	0.001	Accepted
FMS → SCP	0.423	2.036	0.037	Accepted
FMS → BCS	0.127	1.131	0.041	Accepted
FMS → WLB	0.268	1.188	0.031	Accepted
ETT → SEF	0.113	2.584	0.010	Accepted
ETT → SCP	0.407	2.220	0.027	Accepted
ETT → BCS	0.501	2.755	0.006	Accepted
ETT → WLB	0.372	4.994	0.001	Accepted
SEF → BCS	0.201	1.451	0.047	Accepted
SEF → WLB	0.556	2.158	0.031	Accepted
SCP → BCS	0.102	0.523	0.041	Accepted
SCP → WLB	0.295	3.719	0.001	Accepted
Indirect effect				
CLT → SCP → BCS	0.707	3.089	0.002	Accepted
FMS → SEF → BCS	0.208	1.896	0.058	Not Significant
FMS → SEF → WLB	0.325	1.340	0.181	Not Significant
ETT → SEF → BCS	0.613	2.584	0.010	Accepted
ETT → SEF → WLB	0.407	2.220	0.027	Accepted

Source: data analysis result, 2024.

Some of the variables identified as a direct or mediated influence within this structural equation model include culture, family support, entrepreneurial qualities, self-efficacy, social capital, company success, and happiness regarding work-life balance. Independent variables in a direct impact showing an association with their dependent variables presented the following: cultural, family supportive, and entrepreneurial trait variables (CLT, FMS, ETT) all pathways presented *p* < 0.05 and thus were found to be associated statistically. This means that the three variables directly affect business success, work-life balance satisfaction, social capital, and self-efficacy. Two paths, FMS → SEF → BCS and FMS → SEF → WLB, had *p* values of more than 0.05 on the mediation effects, and thus the mediation effects were not statistically significant. That said, self-efficacy may not be a dependable forecaster of both business performance and work-life balance satisfaction.

## 5 Discussion

The results of this analysis provide evidence for the complex relationships between entrepreneurial ventures and culture, family support, entrepreneurial traits, self-efficacy, social capital, business success, and work-life balance satisfaction. The study contributes to a better understanding of the holistic environment in which entrepreneurs operate by explaining the direct and mediating impacts of various variables through diverse lenses.

The important direct impacts that have been noted highlight the critical role of culture, family support and entrepreneurial characteristics in influencing various entrepreneurial outcomes. As a ubiquitous force, culture influences entrepreneurs’ social interactions and self-perceptions in addition to their attitudes and behaviors especially in Indonesia, this is in line with (Gah et al., 2020; Nasihin and Munandar, 2023). Family support is the cornerstone that creates a favorable atmosphere for entrepreneurial ventures, and entrepreneurial qualities act as the spark that drives people to reach for new possibilities and overcome the obstacles that come with being an entrepreneur would be a momentum in the increase of female entrepreneurs in Indonesia. These elements have a major impact on work-life balance happiness, self-efficacy, social capital, and business performance. They are also important in driving general wellbeing and entrepreneurial success (Kadiyono and Yuliafitri, 2023; Anggadwita et al., 2017).

The results of this study complement and extend previous research across a number of important domains related to organizational behavior and entrepreneurship. First, previous research has demonstrated the importance of these variables in influencing entrepreneurial behavior and success (Susanto et al., 2022; Opatrná and Prochazka, 2023). These findings are consistent with the significant direct effects that have been observed between culture, family support, entrepreneurial traits, and various outcome variables, such as. Our findings, which are in line with previous research (Shunmugasundaram, 2022; Siddiqui et al., 2018; Obianefo et al., 2020), highlight the impact of cultural norms, family dynamics, and personal traits on entrepreneurial confidence, social capital, firm prosperity, and satisfaction with work-life balance.

Moreover, investigating mediation effects reveals the intricate mechanisms by which these factors influence each other. There were some discrepancies although most of the mediated relationships showed statistical significance, especially in the paths involving self-efficacy and family support. The insignificant mediation effects highlight the complexity of the relationship between entrepreneurial outcomes and family support, implying that self-efficacy may not be the sole mediator between family support and business success and work-life balance satisfaction (Drnovšek et al., 2024; Agraz-Boeneker and del, 2018). This sophisticated view motivates researchers to take into account the interactions between individual traits, family dynamics, and the larger socio-cultural environment to conduct a deeper investigation into the contextual elements and mechanisms behind this relationship (Prieto-Díeza et al., 2022; Keister et al., 2021).

Moreover, the investigation of mediation effects adds subtlety to our understanding of the mechanisms through which these factors function, which is reinforced by findings from previous studies (Gah et al., 2020; Keister et al., 2021) that examined the mediating functions of social capital and self-efficacy in the relationships between different entrepreneurial antecedents and outcomes. However, the finding of insignificant mediating effects - especially in terms of self-efficacy and family support - extends previous findings by emphasizing how complex these relationships are and pointing to potential boundary conditions or moderators that require more research.

In addition to the influence of entrepreneurial variables on people’s views of work-life balance satisfaction, our findings add to the growing literature on work-life balance and wellbeing such as (Sari et al., 2021; Rahmi et al., 2022). This highlights the need to consider contextual and personal aspects to understand and promote work-life balance among entrepreneurs and is consistent with the increasing recognition of the importance of work-life balance in the context of entrepreneurship (Schiller, 2023; Ng’ora et al., 2022; Samodra et al., 2022).

### 5.1 Theoretical contribution

In sever of ways, this study significantly advances the theoretical groundwork for organizational behavior and entrepreneurship. It begins by presenting a cohesive framework that integrates different theoretical stances from the fields of cultural studies, organizational behavior, and entrepreneurship. An extensive explanation of the entrepreneurial ecosystem is provided by this framework, which explains the linkages between culture, family support, entrepreneurial qualities, self-efficacy, social capital, business success, and work-life balance satisfaction. Second, by performing multilevel analyses, our research expands theoretical understanding beyond the level of the individual. We highlight the significance of taking into account both micro-level characteristics and macro-level influences in influencing entrepreneurial behavior and success by examining the direct and mediated effects of individual, family, and culture factors on entrepreneurial outcomes. Third, by identifying pathways including self-efficacy, social capital, and happiness with work-life balance, this study contributes to theoretical understanding by shedding light on the mediating mechanisms via which cultural, familial, and individual factors effect entrepreneurial success. Furthermore, by highlighting the complex relationships between contextual variables and entrepreneurial outcomes, our research highlights the significance of contextual sensitivity in entrepreneurship theory. The discovery of negligible mediation effects on specific pathways, in turn, draws attention to the presence of border conditions or moderators that affect the link between variables and calls for more research into the contextual elements that moderate these effects. When taken as a whole, these theoretical discoveries deepen our comprehension of the complexity present in entrepreneurship and serve as a foundation for further theoretical growth and empirical research.

### 5.2 Practical implications

These findings have practical consequences for policymakers, educators, and practitioners who encourage entrepreneurial endeavors, beyond the realm of academia. Stakeholders can create focused interventions and activities that improve synergy between individual traits, family support networks, and cultural environments by acknowledging the complex nature of entrepreneurial success and wellbeing. Furthermore, these revelations highlight the significance of adopting a comprehensive strategy that acknowledges the interaction of individual, societal, and environmental elements in promoting the development and resilience of entrepreneurs.

## 6 Conclusion

To sum up, this study advances our knowledge of the intricate relationships that underpin the prosperity and success of entrepreneurs. This research advances theoretical understanding and practical implications by clarifying the direct and intermediary pathways through which culture, family support, entrepreneurial traits, and other factors influence entrepreneurial outcomes. This opens the door to more comprehensive and successful support systems for entrepreneurs in a variety of contexts.

This study’s limitations include its dependence on cross-sectional data, which may make it more difficult to establish causal links, and its absence of several important variables that could have an impact on entrepreneurial outcomes. Self-reported measures have the potential to introduce bias, and the results’ generalizability is constrained by the sample’s lack of variety. To address these limitations, future research should consider longitudinal designs to track changes over time and uncover causal relationships between variables. Such studies could investigate how entrepreneurial qualities evolve and interact with external factors, such as policy changes or economic cycles. Furthermore, examining underlying mechanisms such as the role of emotional intelligence, leadership styles, or gendered experiences in entrepreneurship would deepen our understanding of how various factors collectively influence success.

## Data Availability

The original contributions presented in the study are included in the article/supplementary material, further inquiries can be directed to the corresponding author.
